# Expressed Emotion and Attributions in Parents With Schizophrenia

**DOI:** 10.3389/fpsyt.2021.799626

**Published:** 2021-12-13

**Authors:** Lynsey Gregg, Rachel Calam, Richard J. Drake, Lauren Wolfenden

**Affiliations:** ^1^Division of Psychology and Mental Health, School of Health Sciences, Faculty of Biology, Medicine and Health, Manchester Academic Health Science Centre, The University of Manchester, Manchester, United Kingdom; ^2^Greater Manchester Mental Health NHS Foundation Trust, The University of Manchester, Manchester, United Kingdom

**Keywords:** serious mental illness (SMI), psychosis, child behavior, family environment, warmth, blame

## Abstract

We examined expressed emotion (EE) and attributions in parents with schizophrenia and compared them to parents without serious mental illness (SMI) in order to better understand the emotional climate of families in which a parent has schizophrenia. Parenting practices and parental reports of child behavior were also compared between the two groups. The relationship of EE to attributions was examined in each group separately. Relationships between parental mental health, EE, and attributions were explored in the parents with schizophrenia only. The Camberwell Family Interview was used to determine both EE and attributions in 20 parents with schizophrenia and 20 parents without SMI. We found that more parents with schizophrenia were rated as high EE than those without (60 and 35%, respectively) although this was not a statistically significant difference. Parents with schizophrenia demonstrated significantly more hostility and criticism toward their children than those without SMI and made more child-blaming attributions. Blame was associated with increased hostility, less warmth, and fewer positive remarks. Parental warmth was related to greater parenting self-efficacy, less harsh parenting practices, better child behavior, and a more positive parent–child relationship. We conclude that EE and attributions are potential explanatory variables to be considered in the development of preventative and early intervention strategies for families with a parent with schizophrenia or other psychotic disorder. Blame and warmth are modifiable factors that could be targeted within family and parenting interventions.

## Introduction

Schizophrenia is a chronic and severe illness, with a high global disease burden and significant economic cost (1). The majority of people with psychotic disorders such as schizophrenia are also parents, and their children have been reported to be at significantly increased risk of poor outcomes, including poorer mental health in adulthood (2, 3). Evidence suggests that both genes and environment contribute to an increased risk of intergenerational transmission of schizophrenia (4) and the family environment could plausibly be considered a modifiable environmental factor contributing to this risk.

The importance of the family environment in the development and maintenance of childhood socioemotional and behavioral problems and wellbeing has been well-established in families without a parent with SMI (5–8). There is evidence that dysfunctional and stressful family environments negatively influence parental executive functioning (9) and parenting and family functioning more broadly (10). In families with a parent with a psychotic disorder, who are additionally disproportionately affected by parental unemployment, isolation and poverty (11) these impacts may be even greater. Psychosis has been found to interfere with the establishment and maintenance of important family routines (12) and stigmatization may serve to prevent help-seeking in families that are struggling (13, 14). Ultimately, children of parents with schizophrenia and other psychotic disorders are more likely to be removed from their parents' care (15, 16) with long term consequences for both parent and children's well-being and at significant economic cost for wider society.

Family environments can be explored using the concept of Expressed Emotion (EE) (17). EE is a well-validated measure of the emotional climate of the family which captures the communication style and attitude of a relative when speaking about another family member. Typically assessed by the Camberwell Family Interview (CFI) (17), EE has been widely studied in the family members of individuals with schizophrenia and other psychotic disorders and has been found to be predictive of relapse and hospitalization: Individuals who reside with families characterized by criticism, hostility and emotional-over involvement (“high-EE environments”) are more likely to relapse than those who do not reside in such an environment (18). Conversely, positive affect in the family (characterized by high warmth) has been found to have a protective effect and reduce the likelihood of relapse (19, 20). EE has also been associated with symptoms and functioning in individuals identified as vulnerable to a psychotic disorder (21).

Although, it is plausible that EE may be a potential mechanism of intergenerational transmission in schizophrenia, no research has sought to determine EE in parents living with schizophrenia. This is particularly surprising since parents with mental illness are more likely to have been raised in a family environment characterized by high EE themselves (22). Research to date has primarily focused on families with a depressed parent, finding that EE is typically higher in depressed parents than in those without depression [e.g., (22)] and that in these families, high EE is linked to poorer child behavior [e.g., (23, 24)]. Parental expressed emotion, particularly criticism, has also been associated with the development and maintenance of a range of childhood disorders in families without parental mental illness (25) including increased behavioral problems in individuals with autism (26, 27). Longer terms impacts have also been observed, with parental EE linked to depression, anxiety, and substance misuse in later adulthood (28, 29).

The mechanism of action is not yet established, but the assumption underpinning EE research is that the way parents talk about their relative is indicative of the way they behave toward that relative on a day-to-day basis (30) and causal interpretations of behavior, in the form of attributions, are believed to be the driver of EE (31).

Attributions are beliefs about causality, and are expressions of the way people think about the relationship between an event and a cause (32). Attributional theory suggests that uncovering what people believe about events and their causes is a way to understand and predict their emotional and behavioral responses to those events (33). In the context of parenting, attributions ascribe meaning to children's behavior and guide how the parent relates and responds to their child (34, 35). The key dimensions underlying causal thinking are locus of causality (whether the parent believes the cause of the child's behavior (the “event”) to be internal to or external to the child), controllability (whether the parent considers that the child could control (i.e., prevent) the outcome) universality (whether the cause is personal and unique to the child) and stability (is the cause likely to recur?). Attributions that reveal the parent to consider the cause of an event to be both internal and personal to the child, as well as controllable by the child, are considered “blaming.”

When parents blame a child for behaviors or events their parental responses tend to be more critical and their parenting harsher (23, 24), further contributing to the development and maintenance of child behavioral problems (34, 36, 37).

Exploration of parental EE and attributions may be an effective way of understanding family dynamics in families affected by parental schizophrenia, and EE could potentially be a useful target for early intervention to improve family functioning and improve long term outcomes in these families. Therefore, we explored EE and attributions in parents experiencing schizophrenia, and compared them to parents without serious mental illness (SMI) in the first study of its kind. In line with research conducted with depressed parents we predicted that parents with schizophrenia would demonstrate greater criticism, greater hostility and less warmth toward their children. We also anticipated that they would make more child-blaming attributions and that the frequency of these blaming attributions would be related to EE (specifically, greater criticism and hostility, and less warmth). Relationships between parental mental health, parenting practices, attributions, and EE were also explored in the parents with schizophrenia in order to ascertain whether mental health was associated with facets of EE or attributions and whether EE and attributions had a directly impact on parenting practices.

## Methods

### Ethical Approval

Ethical approval was obtained from Greater Manchester West National Research Ethics Committee.

### Sample

Participants were required to be over 18 years old; a parent/primary care-provider, living with and having direct parenting responsibilities for a child aged between 3 and 11 years. Spoken English was required in order to provide informed consent and complete assessments. Families with multiple children nominated an index child with whom they expressed the greatest parenting challenges. Diagnoses for the parents with schizophrenia were corroborated using International Classification of Diseases (ICD-10) checklists and case note review. Those meeting the criteria for schizophrenia (F20-F29) were eligible. To reduce risk of distress, participants were excluded if they had recently been discharged from in-patient care or if there were known intentions for their child to be removed from their care.

### Recruitment

Recruitment to the clinical group was from four NHS Trusts across Greater Manchester, UK. Community Mental Health Teams and Early Intervention Services were approached. Voluntary sector and social services were also utilized, including Local Authority Family Services. Adverts were also placed on online parenting forums and in schools, local authority services, GP surgeries and nurseries to boost recruitment to both groups. Letters were sent to potentially eligible parents registered on a research volunteer database at the University of Manchester. Participants without SMI self-referred and contacted the research team directly.

### Measures

The Positive and Negative Syndrome Scale (PANSS) (38) and the Psychotic Symptoms Rating Scales (PSYRATS) (39) were used to determine symptom severity in the parents with schizophrenia. The rater established inter-rater reliability after rating ten “gold standard” video-recorded interviews prior to recruitment taking place, achieving an average intraclass correlation coefficient of 0.85. Parental well-being was assessed in both groups using the Warwick Edinburgh Mental Wellbeing Scale (WEMWBS) (40) and negative emotional states were assessed using the Depression, Anxiety and Stress Short Form Scale (DASS-21) (41). Parenting and child behaviors were explored using a range of measures: Parental self-efficacy was explored using the Parenting Task Checklist (PTC) (42). The Parenting and Family Adjustment Scales (PAFAS) (43) assessed parenting strategies and family dynamics and the Parenting Scale (PS) (44) assessed a range of parenting behaviors including the use of permissive (lax) and harsh (over-reactive) approaches. Child behavior was assessed using the Eyberg Child Behavior Inventory (ECBI) (45) which determines intensity and frequency of problematic child behaviors. Alpha levels were in the good to excellent range (α = 0.70–0.95) for all measures except the setting subscale of the parenting task checklist which measures parenting self-efficacy in 14 different settings. Reliability for this subscale was very low at α = 0.20.

#### The Modified Camberwell Family Interview

The original CFI is a standardized semi-structured interview used to assess the emotional attitudes of relatives toward their family member with schizophrenia (17) and is the “gold standard” measure of EE. The modified CFI used in this study was based on previous researchers' adaptations (23, 46). These adaptations focus the CFI on problematic child behaviors as opposed to adult symptom behaviors. Procedures and rating classifications remained unchanged. CFIs were rated for EE by a researcher (LG) who had been formally trained by one of the original developers of the CFI (CV). LG achieved excellent average inter-rater reliability against criterion gold standard raters (0.94). The modified CFI is available from the corresponding author.

The CFI provided ratings of EE on five dimensions: criticism, hostility, EOI, warmth and positive remarks. To rate criticism or “critical comments” statements indicating parental annoyance toward particular behaviors or characteristics are noted and frequency counts collected. Hostility, EOI, and warmth are coded by making conclusions based on information from the entire interview. Hostility is rated when criticism is either generalized or there is rejection and is measured using a four point scale: 0 = no hostility; 1 = generalization only; 2 = rejection only; and 3 = generalization and rejection. EOI uses a six-point-scale with a threshold of 3, and is rated when a parent demonstrates excessive overprotective behaviors or emotional responses toward their child (46). Warmth is an overall rating of sympathy, empathy, interest in and closeness to the child scored from 0 = no warmth to 5 = extreme warmth. Positive remarks reflecting positive parent–child relationships or closeness are noted and frequency counts collected. Parents are classified as “high” EE if there are ≥6 critical comments; ≥3 EOI ratings or hostility is present.

#### Causal Attributions: The Leeds Attributional Coding System

In line with previous research [e.g., (23, 46)] spontaneous parental casual attributions regarding child problem behaviors were extracted from the CFIs using the modified Leeds Attributional Coding System (LACS) (47). The LACS was originally modified by White and Barrowclough (24) for parents experiencing depression. A coding manual created by Peters et al. (46) was adapted for the current study to include examples from parents with schizophrenia. A copy is available from the corresponding author.

Following extraction, attributional statements were coded by the second author and an independent rater (AP) along the four key dimensions included in the LACS: internal/external, controllable/ uncontrollable, personal/universal, and stable/unstable (see Table 1). Statements were coded in accordance with the instructions given in the LACS, which necessitates one rating on each attributional dimension, using a binary scale for each side of the dimensions. A score of 1 was given for the internal, controllable, personal, and stable ends of the four dimensions and a score of 3 was given for the external, uncontrollable, universal, and unstable ends. A score of 2 was assigned when causes appeared to be a mixture of both ends of the dimension (e.g., a cause that was partly controllable and partly uncontrollable by the child).

**Table 1:** Attribution dimensions.

**Dimension**	**Description**
**Internal–external**	Internal: The cause is a “feature” of the child (e.g., personality traits, physical characteristics, illnesses/symptoms, behavior, thoughts, feelings, knowledge, opinions, and beliefs).
	External: Factors outside of or imposed on their child. e.g., Actions or traits of other people, the weather, or location.
**Personal–universal**	Personal: A specific cause leading to an event that would not happen to others (e.g., personality traits, information that identifies that child from others/specific about their child).
	Universal: Expected or understandable behavior for a child of similar age and/or gender (e.g., typical behavior or reactions, conditions).
**Controllable–uncontrollable**	Controllable: Belief that behaviors could be changed, influenced or controlled by child (e.g., Tantrums, sulking, aggression, wanting attention, voluntary behaviors, habits, attitudes, laziness, and irritability).
	Uncontrollable: Belief that that behavior is outside the control of the child, e.g., fear, accidents, illnesses, personality traits/dispositions, characterizes, emotional responses, environmental, or situational factors).
**Stable–unstable**	Stable: The cause as frequent feature or characteristic of the child (e.g., habits or behavior patterns, not sleeping/tiredness, skills, socio-economic difficulties, or life events).
	Unstable: The cause is in past tense or infrequent incidents (e.g., moods, ideas, thoughts, single actions/behaviors, luck-fate, or accidents).

Each attributional statement therefore generated four codes, one for internal/external, one for controllable/uncontrollable, one for personal/universal, and one for stable/unstable. A score of 9 was used in rare cases where the cause could not be rated. For each interview, the total number of attributions made that were rated internal, controllable, and personal to the child were counted (“blaming attributions”). Reliability of extraction and rating between raters (LW and AP) was established on a sample of eight randomly selected CFIs.

Proportional attribution scores indicate the general direction of causality on each dimension and were calculated by dividing the number of causes scored as “1” by the number of causes given a score of 1 or 3 (46). They range between 0 and 1 and scores >0.50 represent attributions that were predominantly internal, controllable, personal, and stable.

### Data Analysis

Data were analyzed using SPSS version 25. EE Criticism and hostility were not normally distributed and were log transformed for analysis. Parents with schizophrenia were compared to parents without SMI using *t*-tests and Chi squared tests. Pearson's *r* correlations were used to assess hypothesized relationships between EE and attributions. Exploratory analyses of relationships between EE, attributions, parental mental health, and parenting also used correlation and *t*-tests. Multiplicity adjustments were not made for these exploratory analyses, despite the large number of tests conducted, in order to avoid accidentally missing true effects (48). Multiple linear regression was used to determine the relative impact of parental mental health status (schizophrenia vs. no SMI) on EE and attributions compared to demographic variables.

## Results

### Participant Characteristics

Participants in the clinical group had diagnoses of schizophrenia (*n* = 11) or paranoid schizophrenia (*n* = 9). Duration of psychosis was 4–5 years (5%), 5–10 years (50%), 11–20 years (30%), and >20 years (15%). Table 2 provides an overview of key demographic characteristics and family circumstances for both groups. Significant differences were observed with regards to parental age, household composition and employment. Parents with schizophrenia were younger [*t*_(38)_ = 2.72, *p* < 0.05] and more likely to be single parents [$X_{\left(1\right)}^{2}$ = 14.55, *p* < 0.001] and unemployed [$X_{\left(2\right)}^{2}$ = 16.04, *p* < 0.001].

**Table 2:** Participant characteristics.

	**Parents with schizophrenia**	**Parents without SMI**
Parent gender, *N* (%)		
Female	19 (95%)	19 (95%)
Male	1 (5%)	1 (5%)
Parent ethnicity, *N* (%)		
White	15 (75%)	16 (80%)
Black	2 (10%)	1 (5%)
Chinese	1 (5%)	0
South Asian	1 (5%)	1 (%%)
Mixed	1 (5%)	2 (10%)
Parent age, mean (SD)	33.9 (7.5)	39.9 (6.4)
Number of children, mean (range)	2 (1–5)	2 (1–6)
Child's gender, *N* (%)		
Female	6 (30%)	7 (35%)
Male	14 (70%)	13 (65%)
Child's ethnicity, *N* (%)		
White	12 (60%)	15 (75%)
Black	1 (%)	1 (5%)
South Asian	1 (5%)	0
Mixed	6 (30%)	4 (20%)
Child's age, *mean (range)*	8 (3–11)	6 (4–10)
Household composition, *N* (%)		
Single parent household	15 (75%)	3 (15%)
Dual parent household	5 (25%)	17 (85%)
Parental employment, *N* (%)		
Unemployed	19 (95%)	7 (35%)
Part time employment	1 (5%)	7 (35%)
Full time employment	0	6 (30%)

### Expressed Emotion: The Modified Camberwell Family Interview

A higher percentage of parents with schizophrenia were rated as high EE overall (*n* = 12, 60%) compared to the non-clinical group (*n* = 7, 35%) although this difference was not found to be statistically significant. They made significantly more critical comments than those without SMI and were more likely to be categorized as highly critical with 50% making 6 or more critical comments compared to 20% of those without SMI [*X*^2^_(1)_ = 3.96, *p* < 0.05]. Parents with schizophrenia were also more likely to be rated as “hostile” with seven (35%) meeting criteria for this rating compared to just one (5%) in the non-clinical group [*X*^2^_(1)_ = 4.33, *p* < 0.05]. Six of these seven parents made “rejecting” comments. For the other dimensions of EE (EOI, warmth, and positive remarks) there were no significant differences between the two groups (see Table 3).

**Table 3:** Differences in expressed emotion, attributions, parenting, and reports of child behavior between groups.

**Expressed emotion**	**Parents with schizophrenia**	**Parents without SMI**	***p***
	**Mean (SD)**	**Mean (SD)**	
EE Criticism^*^	8.3 (7.20)	3.3 (2.25)	0.043
EE Hostility^*^	0.75 (1.21)	0.05 (0.22)	0.021
EE EOI	1.05 (1.05)	1.05 (1.15)	1.00
EE Warmth	1.80 (0.89)	2.25 (0.79)	0.099
EE Positive remarks	2.70 (2.45)	3.30 (1.98)	0.399
Proportional attributions			
Internal	0.55 (0.09)	0.50 (0.17)	0.242
Personal	0.94 (0.08)	0.76 (0.17)	0.000
Controllable	0.86 (0.12)	0.81 (0.10)	0.159
Stable	0.79 (0.20)	0.65 (0.21)	0.032
“Blaming”	0.46 (0.14)	0.33 (0.18)	0.013
Parenting and child behavior			
Behavioral self-efficacy (PTC)	44.17 (22.69)	85.2 (13.32)	0.000
Setting self-efficacy (PTC)	45.52 (25.04)	85.3 (13.76)	0.000
Parental laxness (PS)	4.50 (1.63)	2.35 (0.77)	0.005
Parental reactivity (PS)	3.63 (1.41)	2.54 (0.70)	0.005
Parental verbosity (PS)	4.56 (1.02)	3.91 (0.54)	0.017
PAFAS consistency	8.70 (2.70)	5.10 (2.44)	0.000
PAFAS coercion	7.65 (3.82)	5.90 (3.70)	0.149
PAFAS positive encouragement	5.55 (3.35)	3.80 (3.27)	0.103
PAFAS parent–child relationship	8.35 (2.51)	4.75 (2.81)	0.025
ECBI Intensity	164.55 (44.56)	106.70 (25.04)	0.000
ECBI Problem	21.15 (10.02)	8.35 (8.13)	0.000

* *t-test performed on log transformed variables*.

### Spontaneous Parental Causal Attributions

A total of 950 attributions were extracted from the 40 CFIs (567 from the parents with schizophrenia, 383 from the parents without SMI). The mean rate of attributions per minute indicated that the parents with schizophrenia made more attributions than those without. Table 3 outlines the proportional attribution scores for both groups indicating the predominant direction of causality. Parents with schizophrenia made more attributions that were rated as personal to the child and stable in nature. They also made significantly more “blaming” attributions (attributions rated as internal and personal to, and controllable by, the child).

### Relationship of Demographic Variables to Expressed Emotion and Attributions

Since the parents with schizophrenia differed significantly from those without with regard to age, household composition, and employment status (parents with schizophrenia were younger, more likely to be single parents and less likely to be employed) we conducted multiple linear regression analyses to determine the relative impact of SMI status on the EE and attribution variables where differences between the two groups had been observed. In these regressions age, household composition (single vs. not) and employment status (employed vs. not) were entered in the first step and SMI status (schizophrenia vs. no SMI) was entered in the second. We found that SMI status was not a significant predictor of the two EE variables at the 5% significance level adopted for the study, despite large *R*^2^ values (criticism: *R*^2^ change = 0.09, β = 0.417, *p* = 0.056; hostility: *R*^2^ change = 0.07, β = 0.373, and *p* = 0.096). SMI status predicted personal attributions (*R*^2^ change = 0.09, β = 0.433, *p* = 0.033) but not stable (*R*^2^ change = 0.03, β = 0.239, and *p* = 0.286) or blaming attributions (*R*^2^ change = 0.05, β = 0.320, and *p* = 0.147).

### Parenting and Child Behavior

Table 2 highlights significant differences between the groups with regard to parenting and parental reports of child behavior. Parents with schizophrenia had poorer parenting self-efficacy and were more likely to use harsh (over-reactive), permissive (lax), and overly wordy (verbose) discipline strategies according to their responses to the parenting scale. Parents with schizophrenia were less consistent in their parenting than those without SMI and reported a poorer parent–child relationship. The use of coercion and positive encouragement was not significantly different between groups. Parents with schizophrenia reported significantly more behavior problems in their children (ECBI intensity) and found their children's behavior to be more problematic (ECBI problem).

### Relationship Between EE and Attributions

Correlational analyses (Pearson's *r*) were used to explore hypothesized relationships between EE and attribution variables. Parents with schizophrenia who perceived their children's behavior to be outside of the child's control were warmer about them (*r* = −0.47, *p* < 0.05), and less emotionally over-involved (*r* = −0.69, *p* < 0.001). Parents with schizophrenia who had a tendency to blame the child for the child's negative behaviors were more hostile (*r* = 0.47, *p* < 0.05) and less warm (*r* = −0.60, *p* < 0.01) toward them. However, contrary to our predictions, these parents were not more critical of the child. There were no significant relationships between EE and attributions in the parents without SMI.

### Relationship of Parental Mental Health to EE and Attributions in Parents With Schizophrenia: Exploratory Analyses

The majority of significant relationships between mental health and EE centered on warmth (see Table 4). Higher levels of depression, stress, negative and general symptoms and delusions were each related to decreased warmth. Accordingly, warmth increased as parental subjective wellbeing increased. Higher levels of depression, stress, and anxiety were also related to fewer positive remarks being made. No associations were found between criticism, EOI, hostility and mental health, and well-being. Attribution scores, including blaming attributions, were not related to any mental health variables.

**Table 4:** Correlations between EE, mental health, parenting and child behavior in parents with schizophrenia.

	**Criticism**	**Hostility**	**EOI**	**Warmth**	**Positive Remarks**
DASS depression	0.22	0.31	−0.42	−0.71^**^	−0.62^**^
DASS anxiety	0.25	0.11	−0.42	−0.38	−0.48^*^
DASS stress	0.25	0.31	−0.41	−0.63^**^	−0.62^**^
WEMWBS total	−0.19	−0.12	0.12	0.67^**^	0.47^*^
PANSS positive symptoms	0.23	0.04	−0.20	−0.42	−0.36
PANSS negative symptoms	0.14	0.09	−0.22	−0.53^*^	−0.24
PANSS general symptoms	0.11	0.01	−0.11	−0.47^*^	−0.24
PSYRATS hallucinations	0.05	0.04	−0.07	−0.39	−0.33
PSYRATS delusions	0.42	0.26	0.16	−0.60^**^	−0.43
Setting parental self-efficacy (PTC)	−0.43^*^	−0.41	0.40	0.74^**^	0.57^**^
Behavioral parental self-efficacy (PTC)	−0.23	−0.28	0.24	0.61^**^	0.52^*^
Parental laxness (PS)	−0.14	0.09	−0.08	−0.40	−0.06
Parental reactivity (PS)	0.10	0.45^*^	−0.34	−0.46^*^	−0.02
Parental verbosity (PS)	−0.01	0.11	0.18	0.05	0.18
PAFAS consistency	−0.12	−0.08	0.15	−0.26	−0.33
PAFAS coercion	−0.01	−0.08	0.16	−0.33	0.09
PAFAS positive encouragement	−0.25	−0.06	0.23	0.13	0.34
PAFAS parent–child relationship	0.18	0.31	−0.35	−0.58^**^	−0.29
ECBI Intensity	0.49^*^	0.39	−0.34	−0.62^**^	−0.64^**^
ECBI Problem	0.39	0.44	−0.58^**^	−0.78^**^	−0.34

* *p < 0.05;*

** *p < 0.01*.

*DASS, Depression, Anxiety and Stress Short Form Scale; WEMWBS, Warwick Edinburgh Mental Wellbeing Scale; PANSS, Positive and Negative Syndrome Scale; PSYRATS, Psychotic Symptoms Rating Scales; PTC, Parenting Task Checklist; PS, Parenting Scales; PAFAS, Parenting and Family Adjustment Scales; ECBI, Eyberg Child Behavior Inventory*.

### Relationship of Parenting Practices and Child Behavior to EE and Attributions in Parents With Schizophrenia: Exploratory Analyses

Facets of EE were associated with several aspects of parenting (see Table 4). Critical comments were related to setting specific self-efficacy and ECBI intensity scores. Hostility was related to the use of harsh (reactive) discipline practices and a *t*-test confirmed that parents rated as hostile were more reactive than those who were not [means = 4.60 (1.49) and 3.21 (1.19), respectively, *t*_(18)_ = 2.23, *p* < 0.05]. Emotional over-involvement was related to ECBI problem scores but not to self-efficacy or parenting.

As is clear from Table 4, warmth was once again the facet of EE with the greatest number of significant associations. Lack of warmth was related to reduced parenting self-efficacy and the use of harsher (over reactive) parenting practices. Higher levels of warmth were related to a better parent–child relationship and better child behavior. A similar pattern of results was observed for positive remarks: Increased frequency of positive remarks was associated with reduced efficacy and lower ECBI intensity scores.

There were markedly fewer relationships between proportional attributions and parenting and child behavior. Internal attributions were not related to any parenting or child behavior measures. A tendency to see problems as personal to the child was linked to decreased parental behavioral self-efficacy (*r* = −0.54, *p* < 0.05); problematic behavior (*r* = 0.46, *p* < 0.05) and a poorer parent–child relationship (*r* = 0.46, *p* < 0.05). The perception that the child could control their behavior was also associated with more problematic behaviors (*r* = 0.47, *p* < 0.05) and a poorer parent–child relationship (*r* = 0.57, *p* < 0.01). A tendency to see the causes of behaviors as stable (and likely to recur) was linked to less consistent parenting (*r* = 0.45, *p* < 0.05). Finally, blaming attributions reflected a poorer parent–child relationship (*r* = 0.62, *p* < 0.01) but were not related to specific parenting behaviors otherwise.

## Discussion

This is the first study to examine EE and attributions in parents with schizophrenia, or indeed with any SMI. It confirms that parents with schizophrenia, like other parents, seek to explain their children's behavior and spontaneously make attributions about behaviors they perceive to be negative (24). Furthermore, they do so at a higher rate than parents without SMI. In line with our hypotheses, parents with schizophrenia differed from those without SMI in terms of both expressed emotion and attributions. The finding that parents with schizophrenia were more critical and hostile about their children is an important one, since parental EE has been linked to the development and maintenance of a range of childhood disorders (25) and to mental health difficulties and substance misuse problems in adulthood (28). Our hypothesis that parents with schizophrenia would be less warm than their counterparts without SMI was not supported. This was surprising given the numerous studies reporting poorer parent-infant interactions in mothers with schizophrenia which have highlighted a lack of warmth, sensitivity and responsiveness [e.g., (49–51)]. We did however find that poorer parental mental health was linked to decreased warmth: higher levels of depression, stress and more severe negative and general symptoms and delusions were all associated with lower levels of warmth. It may therefore be the case that the lack of a significant difference between the two groups of parents may in fact reflect symptom variability within the parents with schizophrenia.

Parents with schizophrenia were more likely to attribute their children's behaviors to causes that were more stable and personal to the child and in line with our prediction, they were more likely than the parents without SMI to attribute responsibility for the behavior to the child. This tendency to make more blaming attributions was linked to hostility, which in turn was linked to parenting practices: hostile parents used harsher discipline practices than those who were not hostile. These findings provide support to studies reporting that when parents believe their child's behavior is intentional and unique to the child, they tend to use more coercive and harsh parenting practices (52–54).

Increased blame was also related to a lack of warmth, which in turn was related to reduced parenting self-efficacy and the use of harsher (over reactive) parenting practices. Blame has previously been found to reflect higher levels of parental distress in relation to child behavior (55). Although, we did not assess parents' anger or distress in relation to the child it is indeed likely that this is the driver of a harsher style of parenting. If parents believe that the child is responsible for their behavior, they also believe that the child is capable of modifying it, and parents may therefore engage in more negative feedback (56). The finding that parents with schizophrenia tended to attribute children's behavior to causes that were more stable and personal may additionally indicate a lack of hope for improvements in behavior, further adding to parental distress. It is conceivable that for the parents with the worst mental health, who evidenced the lowest levels of parenting self-efficacy, the tendency to blame children and be less warm toward them may constitute attempts to preserve parental self-esteem and well-being. Future research should aim to further explore the relationship between parental mental health and parental beliefs about children's behavior to elucidate this further.

Owing to the socioeconomic differences between the two groups we considered the possibility that the observed differences in EE and attributions may instead reflect the social isolation and financial stress caused by unemployment and single parenthood. Our regression analyses revealed that this may well be the case. SMI status was not found to independently predict EE although a large effect was observed. This likely reflects the small sample size. A larger sample, with a more closely matched control group would be needed to test this properly. It is likely that the social adversity experienced by parents with schizophrenia is a key feature of the family environment and highly likely to contribute to higher levels of EE toward their children compared to families without SMI who are less likely to be experiencing these stressors. Research shows that families characterized by instability and lacking access to financial resources and social support are families in distress, and this is a key factor in the development of high EE (57).

It must be noted that several unmeasured variables may also have contributed to the observed differences in EE and attributions between the groups, such as comorbid parental and child physical and mental illness.

Despite the study limitations, it is possible to conclude that EE might explain some of the intergenerational risk in families with a parent with schizophrenia. This study highlights opportunities for the development of preventative and early intervention strategies, beyond that of standard family intervention when working with parents with schizophrenia. Parental attributions and EE may offer insight into parenting practices and highlight potential targets for intervention strategies to benefit both parental mental health and longer term outcomes for children.

## Data Availability Statement

The raw data supporting the conclusions of this article will be made available by the authors, without undue reservation.

## Ethics Statement

The study was reviewed and approved by Greater Manchester West National Research Ethics Committee. The patients/participants provided their written informed consent to participate in this study.

## Author Contributions

LG: conceptualization, designing the study, data analysis, writing, reviewing, and editing the manuscript. RC and RD: conceptualization, designing the study, and supervision. LW: conceptualization, designing the study, data collection, and writing the manuscript. All authors contributed to the article and approved the submitted version.

## Funding

LW was supported by a President's Doctoral Scholar award from The University of Manchester.

## Conflict of Interest

The authors declare that the research was conducted in the absence of any commercial or financial relationships that could be construed as a potential conflict of interest.

## Publisher's Note

All claims expressed in this article are solely those of the authors and do not necessarily represent those of their affiliated organizations, or those of the publisher, the editors and the reviewers. Any product that may be evaluated in this article, or claim that may be made by its manufacturer, is not guaranteed or endorsed by the publisher.
